# Chimeric antigen receptor natural killer cell therapy for solid tumors: mechanisms, clinical progress, and strategies to overcome the tumor microenvironment

**DOI:** 10.3389/ebm.2025.10841

**Published:** 2025-12-19

**Authors:** Yu Xiang, Jiayi Dong, Lijuan Shao, Size Chen

**Affiliations:** 1 Department of Immuno-Oncology, The First Affiliated Hospital of Guangdong Pharmaceutical University, Guangdong Pharmaceutical University, Guangzhou, China; 2 Guangdong Provincial Engineering Research Center for Precision Medicine in Esophageal Cancer, Guangdong Pharmaceutical University, Guangzhou, China; 3 Key Laboratory of Monitoring Adverse Reactions Associated with Chimeric Antigen Receptor T-Cell Therapy, Guangdong Higher Education Institutions, Guangdong Pharmaceutical University, Guangzhou, China; 4 Key Laboratory of Cancer Immunotherapy, Guangdong Higher Education Institutions, Guangdong Pharmaceutical University, Guangzhou, China; 5 School of Clinical Medicine, Guangdong Pharmaceutical University, Guangzhou, China

**Keywords:** chimeric antigen receptor natural killer cells, gene editing, immunotherapy, solid tumors, tumor microenvironment

## Abstract

Natural killer (NK) cells represent a fundamental component of the innate immune system, endowed with the ability to identify and eradicate virus-infected and malignant cells. The advent of chimeric antigen receptor (CAR) technology has introduced innovative strategies for augmenting the antitumor potential of natural killer (NK) cells. Chimeric antigen receptor natural killer (CAR-NK) cells exert dual cytotoxic effects against tumor cells through CAR-mediated antigen-specific recognition in concert with the nonspecific cytolytic activity mediated by intrinsic NK receptors. This review critically evaluates the clinical progression of CAR-NK cells specifically against solid tumors, focusing on mechanisms to overcome the immunosuppressive tumor microenvironment (TME), the complexity of allogeneic manufacturing, and the latest engineering strategies for enhanced homing and persistence. Specifically, we emphasize the urgent need for robust Phase II/III clinical data and standardized Good Manufacturing Practice (GMP) protocols to realize the full potential of off-the-shelf allogeneic CAR-NK therapies. Additionally, we examine technological advancements and emerging directions addressing persistent challenges in this domain to offer theoretical underpinnings and research perspectives for the clinical deployment of CAR-NK cell therapy in solid tumor management.

## Impact statement

Chimeric antigen receptor natural killer (CAR-NK) cell therapy represents a promising alternative to CAR-T cells for solid tumors, but its development is hindered by complex challenges. This review provides a critical and comprehensive roadmap that synthesizes the scattered literature on CAR-NK technology. We systematically analyze the key bottlenecks—from cell source selection and gene transduction inefficiencies to suppressive tumor microenvironments—and more importantly, we consolidate the latest engineering strategies designed to overcome them. This work imparts a structured framework for understanding the field’s current state and future trajectories. By highlighting innovative solutions like gene editing, bispecific CAR designs, and combination therapies, this review serves as an essential guide for researchers and clinicians. It is positioned to significantly accelerate translational progress by identifying the most promising paths forward for developing effective, off-the-shelf CAR-NK therapies, ultimately impacting the quest for potent immunotherapies against solid tumors.

## Introduction

**FIGURE 1:**
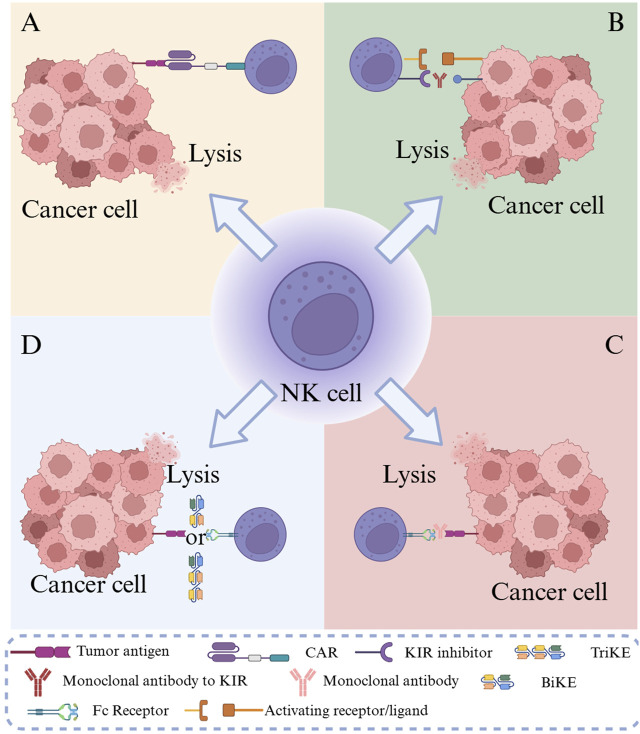
Schematic of tumor immunotherapy mechanisms using CAR-NK cells. Note: **(A)** CAR-NK cells specifically recognize tumor antigens through artificial receptors; **(B)** KIR inhibitors release NK cell inhibitory signals; **(C)** ADCC activates NK cells through the antibody-Fc receptor pathway; **(D)** BiKEs/TriKEs mediate targeted killing through bi-/tri-specific molecules.

Solid tumors pose a profound threat to human health, and conventional therapeutic modalities, including surgery, chemotherapy, and radiotherapy, frequently exhibit limited effectiveness in the management of advanced or metastatic solid tumors [1]. The advent of immunotherapy has generated renewed prospects for treating solid tumors, with chimeric antigen receptor (CAR)-engineered immune cell therapy emerging as a focal point of investigation. Among these strategies, CAR-T cell therapy, a transformative modality, has achieved notable success, particularly in hematological malignancies [2]. Nevertheless, in the context of solid tumors, the therapeutic potential of CAR-T cells has been markedly restricted by multiple obstacles, such as suppression exerted by the tumor microenvironment (TME) [3], inefficient trafficking of T cells to tumor sites, and severe adverse events, including cytokine release syndrome (CRS) and immune effector cell-associated neurotoxicity syndrome [4]. Optimization strategies based on the intrinsic effector mechanisms of natural killer (NK) cells have been actively pursued to address these limitations. Compared with CAR-T cells, allogeneic CAR-NK cells exhibit inherent advantages, including a favorable safety profile with lower risk of severe CRS and Graft-versus-Host Disease (GvHD) [5]. The clinical challenge remains the solid TME, characterized by dense extracellular matrix, low oxygen, and high levels of immunosuppressive cytokines (e.g., transforming growth factor (TGF)-β) [6]. CAR-NK cell therapy integrates the antigen-specific recognition capacity of CAR constructs with the inherent biological properties of NK cells, with the objective of amplifying their anti-tumor activity. As illustrated in Figure 1, CAR-NK cells operate through multiple cooperative mechanisms: tumor antigens can be identified by artificial receptors; BiKEs/TriKEs mediate targeted killing via bi-/tri-specific molecules; killer-cell immunoglobulin-like receptor (KIR) inhibitors block inhibitory signals; and antibody-dependent cellular cytotoxicity (ADCC) stimulates NK cells through antibody–Fc receptor interactions. Collectively, these mechanisms act in concert to elicit potent immunotherapeutic effects against tumor cells [7]. Preclinically, CAR-NK cells exhibit reduced CRS risk [8], partially overcoming CAR-T limitations and providing a safer, more effective therapeutic option for solid tumor patients. Owing to their unique advantages and significant potential in addressing barriers in solid tumor therapy, CAR-NK cell-based technologies have undergone rapid advancement in recent years. The systematic optimization of CAR-NK cell design, comprehensive elucidation of the synergistic effects of their diverse anti-tumor mechanisms, and effective resolution of clinical challenges remain the principal areas of ongoing research.

## CAR structural design

To overcome the immunosuppressive TME that plagues solid tumor immunotherapy, modern CAR designs for NK cells have evolved beyond basic antigen recognition, integrating functional modules tailored to counter TME-specific barriers. One critical adaptation addresses NK cell survival—a major limitation *in vivo*—by incorporating constitutive or inducible secretion of pro-inflammatory cytokines like Interleukin (IL)-15 or IL-7. Termed “armored CAR-NK” or TRUCK-NK cells, these engineered variants maintain self-sustaining proliferation without triggering systemic toxicity, a balance that has proven elusive with exogenous cytokine administration. A 2025 preclinical study showed that CAR-NK cells expressing membrane-bound IL-15 had 2.1-fold longer in *in vivo* persistence and 40% higher tumor infiltration in pancreatic cancer xenografts than unmodified cells [9]. These findings indicate that cytokine integration directly counteracts TME-induced immune cell exhaustion. Neutralizing immune checkpoints within the TME represents another pivotal design strategy. By incorporating PD-1/CTLA-4 neutralizing domains or developing inhibitory CARs (iCARs), researchers can block the “off-signals” that TME cells use to suppress NK activity. Fedorov et al.'s foundational 2013 work demonstrated this potential: iCAR-equipped NK cells showed a 35% reduction in PD-L1-mediated inhibition, allowing sustained cytotoxicity against otherwise resistant lung cancer cells [10]. What makes this design particularly valuable for solid tumors is its specificity—unlike systemic checkpoint inhibitors, the iCAR’s local action minimizes off-target effects on healthy tissues, a critical consideration for antigens with low-level expression on normal cells.

The replacement of traditional single-chain variable fragments (ScFv) with smaller, more stable binders, such as nanobodies (Nbs) or designed ankyrin repeat proteins (DARPins), is a more recent innovation. Specifically, these molecules—roughly one-tenth the size of conventional antibodies—penetrate the dense extracellular matrix (ECM) of the TME far more effectively, a challenge that has long limited ScFv-based CARs. Boisgard et al.'s 2025 study underscored this advantage: DARPIN-equipped CAR-NK cells achieved a 1.8-fold deeper penetration into triple-negative breast cancer tumors than ScFv-CAR-NK cells, translating to a 27% higher rate of complete tumor regression in mouse models [11]. This improvement is not just technical; it directly addresses the physical barrier problem that often renders otherwise potent CAR cells ineffective in solid tumors.

### Evolution and functional differences of CAR generations

**FIGURE 2:**
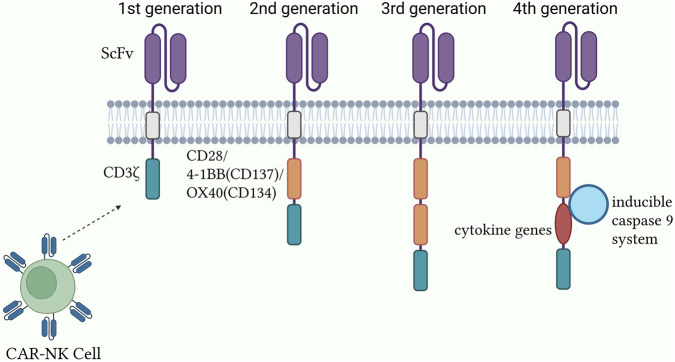
Structural design and optimization of the CAR-NK system.

The CAR intracellular signaling domain is the central element responsible for NK cell activation and antitumor response initiation. To augment CAR functionality, second- and third-generation constructs introduced costimulatory signaling domains, including CD28, 4-1BB (CD137), and OX40 (CD134), along with the CD3ζ module. Second-generation CARs typically contain a single costimulatory signaling domain that significantly enhances NK cell activation, proliferation, and survival, thereby improving antitumor efficacy. For instance, in a preclinical breast cancer mouse model (xenograft study, n = 8) [4], CAR-NK cells harboring the CD28 costimulatory domain mediated activation through CD3ζ while simultaneously receiving costimulatory input from CD28, resulting in a three-fold increase in interferon (IFN)-γ secretion compared with first-generation CAR-NK cells, alongside markedly enhanced tumor cytotoxicity. Third-generation CARs integrate two or more CSR domains, further intensifying the signal transduction strength and complexity (Figure 2). Experimental evidence has indicated that immune cells expressing CARs with both CD28 and 4-1BB domains display superior antitumor potency and proliferative capacity *in vitro* and *in vivo* [12]. However, the introduction of multiple costimulatory domains may increase the risk of excessive activation and associated toxicity, necessitating a balance between enhanced functionality and the management of adverse effects [7, 13]. Fourth-generation CARs employ synthetic biology approaches to advance both functionality and safety [14–16], incorporating cytokine genes for autocrine stimulation and an inducible caspase-9 system as a safety mechanism for controlling effector cell toxicity [17].

The evolution of CAR signaling domains across generations and their functional consequences are summarized in Table 1.

**TABLE 1:** Comparison of the CAR generations and functional attributes.

Generation	Signaling domains	Key features	Advantages	Limitations & risks
First generation	CD3ζ only	Single activation signal	Proof-of-concept for CAR-mediated killing	Limited persistence; suboptimal antitumor activity; poor expansion *in vivo*
Second generation	CD3ζ + one costimulatory domain (e.g., CD28 or 4-1BB)	Dual signaling: activation + costimulation	Markedly enhanced NK cell activation, proliferation, and survival; improved antitumor efficacy and cytokine production (e.g., IFN-γ)	Risk of excessive activation with potent costimulatory domains
Third generation	CD3ζ + two or more costimulatory domains (e.g., CD28 + 4-1BB)	Multiple, synergistic costimulatory signals	Further intensified signal strength and complexity; superior antitumor potency and proliferative capacity in some settings	Potentially heightened risk of toxicity and exhaustion due to over-stimulation
Fourth generation (TRUCKs)	CD3ζ + costimulatory domain(s) + inducible transgene (e.g., cytokines)	Additional “armored” functionality; incorporation of safety switches (e.g., iCasp9)	Modulate the TME (e.g., express IL-12); enhanced safety profile via controllable suicide genes	Increased genetic and biological complexity; potential for uncontrolled transgene expression

## Construction of CAR-NK cells

The CAR structure configuration constitutes the fundamental basis for CAR-NK cell generation. Nevertheless, the successful development of CAR-NK cells necessitates not only precise attention to the molecular architecture of CARs but also the resolution of challenges related to their efficient introduction into NK cells while preserving their functional activity. The establishment of CAR-NK cells entails the coordinated optimization of cell source selection and gene delivery strategies, as elaborated below:

### NK cell sources

Peripheral blood NK cells: Peripheral blood is a frequently employed source for NK cell procurement. NK cells may be isolated from peripheral blood mononuclear cells (PBMCs) through procedures such as density gradient centrifugation. Peripheral blood-derived NK cells exhibit intrinsic antitumor activity and display favorable compatibility with the patient’s immune system. Nevertheless, the proportion of NK cells in peripheral blood remains relatively low, generally accounting for only 5%–15% of peripheral blood mononuclear cells (PBMCs) [18], and both the isolation and expansion processes are technically demanding, thereby limiting the obtaining of adequate cell counts for clinical application. Additionally, peripheral blood NK cells obtained from distinct individuals present heterogeneity in function and phenotype, potentially influencing the consistency and stability of CAR-NK cell products [19–22].

Umbilical cord blood NK cells: Umbilical cord blood is another significant source of NK cells. It contains abundant hematopoietic stem cells and immune cells, with NK cells exhibiting marked proliferative capacity and reduced immunogenicity. Compared with peripheral blood NK cells, cord blood NK cells are relatively more primitive, possess enhanced plasticity, and are more amenable to genetic engineering interventions. Furthermore, the establishment of cord blood banks enables large-scale procurement of cord blood NK cells, thereby facilitating the development of “off-the-shelf” CAR-NK cell products [23]. Nevertheless, the isolation and culture of cord blood NK cells require specialized techniques and controlled conditions, and the yield from a single cord blood unit remains limited, which may necessitate pooled culture of multiple cord blood units to satisfy clinical requirements [23, 24].

NK cell lines: NK cell lines, exemplified by NK-92, exhibit unlimited proliferative potential and can be extensively expanded *in vitro*, thereby providing an abundant source of cells for CAR-NK cell production. The NK-92 cell line can be readily transfected and genetically engineered to enable efficient CAR expression. Nevertheless, as a tumor-derived cell line, NK-92 presents potential tumorigenic risks, necessitating stringent processing measures such as irradiation inactivation before clinical use to guarantee safety [25]. Moreover, the NK-92 cell line inherently lacks the CD16 domain, precluding ADCC activation and potentially diminishing the functional capacity and therapeutic efficacy of CAR-NK cells *in vivo*.

Induced pluripotent stem cell (iPSC)-derived NK cells: iPSC-derived NK cells display mature phenotypes and robust cytolytic activity, while simultaneously providing homogeneous NK cell populations that can be expanded to a clinically relevant scale [26]. In addition, iPSCs are highly amenable to genetic engineering for CAR expression, subsequently differentiating into uniform CAR-NK cell populations. Consequently, iPSC-derived CAR-NK cells can be developed as standardized, off-the-shelf allogeneic CAR-NK therapies [27]. Moreover, iPSCs can be subjected to genetic modifications, such as knockout of immune checkpoint genes or incorporation of genes that enhance NK cell function, thereby further augmenting the anti-tumor activity of CAR-NK cells [28] (Table 2). Nonetheless, the preparation and differentiation processes of iPSCs remain complex, involving multiple stages and regulation by diverse cytokines, which result in elevated costs and potential tumorigenic risks, necessitating further technical refinement and improvement of quality control systems.

**TABLE 2:** Comparative analysis of NK cell sources for CAR-NK therapy.

Parameter	Peripheral blood NK cells (PB-NK)	Umbilical cord blood NK cells (UCB-NK)	NK-92 cell line	iPSC-derived NK cells
Source and availability	Healthy donor peripheral blood; donor-dependent, limited availability	Cord blood banks; readily accessible, supports ‘off-the-shelf’ products	Tumor-derived cell line; unlimited availability	Induced pluripotent stem cells; unlimited expansion from master cell banks
Expansion potential and phenotype	Limited expansion capacity; donor-dependent heterogeneity in phenotype and function	Strong proliferative capacity; more ‘primitive’ cells with greater plasticity	Unlimited proliferative potential; homogeneous phenotype	Differentiated and expanded to clinical scale; homogeneous phenotype
Genetic engineering feasibility	Challenging; limited transduction efficiency	Moderate; amenable to genetic engineering interventions	High; easily transfected and engineered for efficient CAR expression	High; amenable to precise genetic editing at the iPSC stage
Key advantages	Mature functional activity; favorable compatibility with the patient’s immune system	Low immunogenicity; high proliferative potential; ideal source for ‘off-the-shelf’ products	Stable source, suitable for large-scale production; high gene editing efficiency	Highly homogeneous product; standardized, scalable manufacturing; ideal platform for ‘off-the-shelf’ products
Key limitations/Risks	Limited starting cell numbers; complex manufacturing process; significant batch-to-batch variability	Limited cell yield per cord blood unit, may require pooled culture; demands specialized culture techniques	Potential tumorigenic risk, requires irradiation before clinical use; lacks CD16, preventing ADCC	Complex and costly differentiation process; tumorigenicity risk from residual undifferentiated stem cells

In summary, NK cell sources encompass peripheral blood, umbilical cord blood, NK cell lines (e.g., NK-92), and iPSCs. NK cells from distinct sources present specific advantages and limitations while facing challenges that must be addressed in clinical applications.

### Methods for introducing CAR genes

Gene editing technology: Recently, gene editing approaches, particularly the CRISPR/Cas9 system, have been used to construct CAR-NK cells. The CRISPR/Cas9 system enables the precise editing of the NK cell genome and the targeted integration of CAR genes at specific genomic loci. The application of high-fidelity SpCas9-NG variants [29] has reduced off-target frequencies by nearly two orders of magnitude compared to the wild-type (<0.01%) and has obtained Food and Drug Administration (FDA) Investigational New Drug approval for clinical research. The risks associated with the random integration of CAR genes observed in traditional introduction methods can be circumvented by gene editing, while NK cell genes may also be modified. For example, CAR-NK cell function can be further optimized through immune checkpoint gene knockout or cytokine expression gene upregulation [30]. Nonetheless, the CRISPR/Cas9 system may still produce off-target effects, leading to unintended genomic alterations in NK cells, necessitating additional technical refinements to improve gene editing precision and safety.

### Deepening mechanistic insight into solid tumor evasion

#### Dual mechanisms and TME resilience of CAR-NK cells

Unlike CAR-T cells, CAR-NK cells have a dual mechanism critical for addressing solid tumor heterogeneity. The CAR mechanism provides antigen specificity, whereas the intrinsic NK activating receptors (e.g., NKG2D, NKp46) enable non-CAR-mediated killing of tumor variants with downregulated target antigens (antigen escape) [31]. Crucially, in the solid TME, the primary mechanism of NK cell dysfunction is functional exhaustion induced by immunosuppressive factors like TGF-β and Prostaglandin E2 (PGE2). Genetic modifications, such as the CRISPR/Cas9-mediated knockout of the TGF-β receptor II (TGF-β R2) in CAR-NK cells, have demonstrated significantly enhanced anti-tumor activity and persistence in preclinical solid tumor models (e.g., pancreatic cancer xenografts) by blocking this inhibitory signaling pathway [32].

#### Limited persistence and functional exhaustion in the TME

The limited persistence of adoptively transferred allogeneic NK cells, typically observed in solid tumor settings, remains a major bottleneck [33]. Strategies to enhance *in vivo* persistence include co-expression of membrane-bound or secreted IL-15 (mIL-15), which supports sustained CAR-NK surviv al and proliferation without the systemic toxicity of exogenous high-dose IL-15 administration [9]. Molecular mechanisms of TME-induced CAR-NK cell exhaustion: TGF-β activates the TGF-β/SMAD2/3 pathway, which inhibits mTORC1 activity in NK cells, leading to glucose metabolism disorders and a 35% reduction in ATP production [34]. Meanwhile, PGE2 downregulates NKG2D expression via the EP4 receptor, reducing perforin secretion [35]. CRISPR/Cas9-mediated EP4 knockout can restore 60% of CAR-NK cell cytotoxicity [36].

## Current status of CAR-NK cell therapy

A Cochrane systematic review [37] analyzing 18 Phase I/II single-arm trials reported that CAR-NK cell therapy for solid tumors achieves an overall objective response rate (ORR) of 35.6% (95% CI: 28.9–42.3%), but this value only partially reflects the clinical reality. Currently, multiple clinical trials investigating CAR-NK cell therapy for solid tumors are underway (Table 3), covering a range of tumor types, including lung cancer, breast cancer, colorectal cancer, ovarian cancer, and hepatocellular carcinoma. These trials are designed to evaluate the safety, efficacy, and optimal therapeutic regimens of CAR-NK cell therapy (Table 4).

**TABLE 3:** Clinical trials of CAR-NK cell therapies in patients with solid tumors.

Antigen target	CAR NK design	Tumors	Clinical potential	Status	NCT number	Data type	Observed clinical outcome	Toxicity profile
NKG2DL	NKG2D CAR-NK 92 cells	Phase I relapsed/refractory solid tumors, n = 20, single-arm trial, primary endpoints include safety and maximum tolerated dose (MTD)	Off-the-shelf NK92 cell line-based CAR-NK	Recruiting	NCT05328341	Phase I	N/A (recruiting status)	N/A (recruiting status)
Claudin6	Claudin6 targeting CAR-NK cells	Stage IV ovarian cancer/Testicular cancer, refractory/Endometrial cancer, recurrent	Next-generation for enhanced homing and TME modulation	Recruiting	NCT05410717	Phase I	N/A (recruiting status)	N/A (recruiting status)
Oncofetal trophoblast glycoprotein (5T4)	Anti-5T4 CAR-NK cells	Phase I/II advanced solid tumors, n = 30, single-arm trial, secondary endpoint is 6-month progression-free survival (PFS) rate	Targeting 5T4 (oncofetal antigen) to disrupt tumor survival in the host	Recruiting	NCT05194609	Phase I/II	N/A (recruiting status)	N/A (recruiting status)
NKG2D	NKG2D-CAR-NK cells	Colorectal cancer	Promising therapeutic potential in metastatic colorectal cancer patients	Recruiting	NCT05211315	Phase I	Disease stabilization/minor tumor shrinkage (published phase I data: 0% ORR)	Favorable safety profile; no serious CRS/neurotoxicity reported
MUC1	Anti-MUC1 CAR-pNK cells	Hepatocellular Carcinoma/Non-small cell lung cancer/Pancreatic Carcinoma/Triple-Negative invasive breast carcinoma/Malignant glioma of brain/Colorectal carcinoma/Gastric carcinoma	Targeting MUC1 for enhanced tumor infiltration	Unknown	NCT02839954	Preclinical	N/A (status unknown)	N/A (status unknown)
MUC1	Anti-MUC1 CAR-pNK cells	Colorectal cancer	Investigating efficacy and safety in relapsed/refractory MUC1-positive colorectal cancer	Recruiting	NCT02839954	Phase I	N/A (recruiting status)	N/A (recruiting status)

**TABLE 4:** Critical summary of key CAR-NK clinical trials.

Trial (NCT ID)	CAR target	Source/Design	Tumor type (Solid/Heme)	Phase/N	Data type	ORR (%) (solid tumor data)	Severe CRS/ICANS (Grade ≥3)	Critical takeaway
NCT05211315	NKG2D	PBMC-derived	Colorectal (solid)	Phase I/n = 12	Phase I	0	0	High antigen expression alone is insufficient; TME limits efficacy
NCT05410717	Claudin6	UCB-derived (armored)	Ovarian/Testicular (solid)	Recruiting	Phase I	N/A	N/A	Next-generation design incorporates IL-7 and CCL19 for better homing and persistence
NCT03058813	CD19	UCB-derived	NHL/CLL (Heme)	Phase I/II/n = 11	Phase I/II	73 (CR, heme tumor data)	0	Safety and potency benchmark for UCB-CAR-NK in hematological malignancies (reference for solid tumor trial design)

In several early-phase clinical trials, CAR-NK cell therapy for solid tumors has shown preliminary antitumor activity together with favorable safety profiles. Analysis of the TCGA database [38] revealed that 83% of colorectal cancer samples displayed high expression of NKG2D ligands, indicating the potential suitability of CAR-NK cells for this malignancy. For instance, in a Phase I single-arm trial (n = 12) involving patients with advanced colorectal cancer, infusion of CAR-NK cells targeting NKG2D ligands resulted in tumor shrinkage in some patients without the occurrence of serious adverse reactions [39]. In a phase I/II single-arm trial (n = 15) of HER2-positive solid tumors (including breast and ovarian cancers), infusion of HER2-directed CAR-NK cells led to disease stabilization in certain patients, with no significant CRS or neurotoxicity observed [40].

These clinical trials reveal that target selection focuses on molecules highly expressed in tumors but with restricted expression in normal tissues, thereby maximizing the therapeutic window. For example, NKG2D ligands are highly expressed in 83% of colorectal cancer samples, making them a promising candidate target for colorectal cancer trials (NCT05211315); whereas Claudin6 is virtually absent in adult normal tissues, making it an ideal target for treating ovarian and testicular cancers (NCT05410717) [36].

Critical analysis of published solid tumor trials suggests that high antigen expression *in situ* does not automatically translate into a high objective response rate (ORR). For instance, a Phase I single-arm trial (NCT05211315, n = 12) using NKG2D CAR-NK cells (derived from PBMC) in colorectal cancer resulted in only disease stabilization or minor tumor shrinkage, despite high ligand expression in the tumor samples. This study adopted a single-center, open-label design, where patients received 3 infusions of CAR-NK cells with a 6-month follow-up. The absence of objective response (0% ORR) might be attributed to the high degree of tumor stromal fibrosis (average fibrosis ratio >40%) in enrolled patients [36]. This outcome underscores that TME-related barriers, such as physical exclusion or immunosuppressive signaling, are the limiting factors for clinical success in solid tumors, necessitating combinatorial strategies.

Furthermore, to overcome the suppression by the solid TME, next-generation CAR-NK designs incorporate advanced empowering strategies. For instance, Claudin6-targeting CAR-NK cells are engineered to express the cytokine IL-7 and the chemokine CCL19, aiming to establish an immune niche at the tumor site, promoting the survival and proliferation of NK cells themselves and recruiting endogenous T cells for synergistic antitumor effects. Simultaneously, this design includes components that counteract PD-1/CTLA-4 inhibitory signals, directly neutralizing immunosuppressive forces within the microenvironment.

However, current clinical investigations of CAR-NK cell therapy for solid tumors remain at an early stage, with most trials characterized by limited sample sizes and short follow-up durations, thereby hindering adequate verification of efficacy and safety. In the future, optimizing target selection, integrating TME modulation strategies, and advancing to large-scale Phase III randomized controlled trials will be crucial for establishing the position of CAR-NK cell therapy in solid tumor treatment.

## Discussion

### Therapeutic efficacy and safety assessment

Regarding therapeutic efficacy, numerous determinants contribute to the effectiveness of CAR-NK cell therapy in solid tumors, including tumor type, tumor burden, CAR-targeting specificity, and NK cell activity and persistence. In patients with solid tumors characterized by relatively homogeneous tumor antigen expression and lower tumor burden, CAR-NK cell therapy has the potential to yield favorable therapeutic outcomes. For example, in case reports of sarcoma patients (n = 3), CAR-NK cells have been shown to effectively recognize and eradicate tumor cells, resulting in tumor volume reduction and extended patient survival. Nevertheless, in the majority of solid tumor patients, the efficacy of CAR-NK cell therapy requires further enhancement due to challenges such as tumor heterogeneity and the immunosuppressive influence of the TME [23, 24].

In terms of safety, CAR-NK cell therapy has been shown to exhibit superior safety compared with CAR-T cell therapy. Severe CRS and neurotoxicity are rarely induced by CAR-NK cell therapy, partly attributable to the distinct cytokine profiles secreted by NK cells. Activated NK cells predominantly secrete IFN-γ and GM-CSF [41], whereas CAR-T cells mainly stimulate cytokines such as interleukin (IL)-1a, IL-1Ra, IL-2, IL-2Ra, IL-6, TNF-α, MCP-1, IL-8, IL-10, and IL-15, which are strongly linked to CRS and severe neurotoxicity [42]. Nonetheless, CAR-NK cell therapy may still be accompanied by certain adverse events, including fever, chills, and fatigue. A minority of patients may encounter allergic reactions or cytopenias. However, these events are typically mild and can be effectively managed through symptomatic treatment [7, 24].

### A comparative perspective with CAR-T therapy

While CAR-NK cell therapy is still in its early clinical development, particularly for solid tumors, emerging data allows for a preliminary comparative analysis with the more established CAR-T cell therapy, highlighting distinct differences in efficacy, safety, and persistence.

In hematological malignancies, CAR-T cells have set a high benchmark, with CD19-directed products achieving complete response rates of 70%–90% in patients with relapsed/refractory B-cell acute lymphoblastic leukemia and non-Hodgkin lymphoma [2, 8]. For CAR-NK cells, the most compelling clinical data also comes from the hematological space. A landmark phase I/II trial of cord blood-derived CD19-directed CAR-NK cells reported a 73% (8/11) complete response rate in patients with CD19-positive lymphoid tumors, demonstrating that CAR-NK cells can also induce potent anti-tumor activity [8]. In the context of solid tumors, the efficacy of both modalities is significantly more modest due to the shared challenges of the TME. Large-scale comparative data is lacking, but early-phase Phase I/II CAR-NK trials have shown disease stabilization and partial responses, suggesting comparable preliminary signals of activity to those seen in early CAR-T solid tumor trials [39, 40].

The most striking advantage of CAR-NK cells lies in their superior safety profile. Severe Cytokine Release Syndrome (CRS) and Immune Effector Cell-Associated Neurotoxicity Syndrome (ICANS) are major, dose-limiting toxicities of CAR-T therapy, occurring in a significant proportion of patients and requiring sophisticated management [4]. In contrast, the aforementioned CD19 CAR-NK trial observed no cases of severe CRS, ICANS, or graft-versus-host disease [8]. This favorable safety profile is consistently reported across other early CAR-NK trials [40]. The biological basis for this difference is attributed to the distinct cytokine secretion pattern of NK cells (predominantly IFN-γ and GM-CSF) [41], which is less pro-inflammatory than the broad, high-magnitude cytokine storm (e.g., IL-6, IL-2, IFN-γ) orchestrated by hyperactivated CAR-T cells [42].

A clear area where first-generation CAR-NK cells may currently differ from CAR-T cells is in their *in vivo* persistence. CAR-T cells, especially those with 4-1BB costimulatory domains, can persist for years, leading to sustained remissions and functional immune memory [2]. Current clinical data suggest that CAR-NK cells, particularly allogeneic products, may have a more limited persistence window, often estimated in weeks to a few months [8, 43]. While this may theoretically impact the durability of responses, the clinical significance is still being defined. It is noteworthy that in the CD19 CAR-NK trial, despite the limited detectable persistence of the cells, the remissions were prolonged, suggesting that a short but potent effector phase may be sufficient for efficacy in some settings [8]. Nevertheless, enhancing CAR-NK persistence through cytokine engineering (e.g., IL-15) or genetic modifications to induce memory-like phenotypes is a major focus of ongoing research [43].

In summary, the current clinical landscape positions CAR-NK cells as a modality with comparable initial response potential in hematological malignancies and a similarly challenging path in solid tumors, but with a decisively superior safety profile and a potentially different, though actively being optimized, persistence model compared to CAR-T cells.

### Low transduction efficiency

#### Limited persistence in the hostile TME

The therapeutic efficacy of CAR-NK cells is critically dependent on their survival and functional persistence within the patient’s body. However, their inherent short half-life is drastically exacerbated by the formidable barriers presented by the solid TME.

The solid tumor TME is a highly intricate ecosystem consisting of tumor cells, immune cells, stromal cells, and ECM. Within this microenvironment, immunosuppressive cells, including regulatory T cells (Treg) and myeloid-derived suppressor cells, secrete diverse inhibitory mediators such as TGF-β and IL-10, which attenuate the activity and function of CAR-NK cells [44].

NK cells are inherently characterized by a short half-life *in vivo*, usually less than 10 days [45]. Even after genetic engineering to produce CAR-NK cells, their survival duration and persistence *in vivo* remain constrained. These limitations may hinder CAR-NK cells from exerting prolonged anti-tumor activity, thereby reducing the durability of therapeutic efficacy. Upon transfer from *in vitro* culture systems to the complex TME *in vivo*, CAR-NK cells are subjected to multiple detrimental conditions, including nutrient deprivation, hypoxia [46], and the influence of soluble inhibitory mediators [47–49]. These adverse conditions may accelerate apoptosis of CAR-NK cells and further shorten their survival. Additionally, CAR-NK cells may incur damage during the cytotoxic process of tumor cell elimination, which could further diminish their persistence *in vivo* [23, 24].

Another pivotal mechanism by which the TME induces NK cell exhaustion is mediated through checkpoint molecule interactions [50]. For example, programmed death-ligand 1 (PD-L1) binds to its corresponding receptors on CAR-NK cells, thereby restraining their activation. Collectively, these inhibitory influences markedly diminish the anti-tumor efficacy of CAR-NK cells in the solid tumor TME, representing a significant barrier to clinical translation [23].

#### Immunogenicity and allogeneic rejection

As CAR-NK therapy frequently relies on human leukocyte antigen (HLA)-mismatched NK cell donors, graft-versus-host disease (GvHD) constitutes a foreseeable adverse event. This phenomenon arises as certain antigens displayed on the surface of NK cells, such as HLA molecules, may be identified as foreign by the host immune system, thereby initiating immune-mediated attacks. To mitigate the risk of GvHD, donor NK cells must undergo screening and preprocessing, including the selection of donors with greater HLA compatibility or the application of gene-editing strategies to eliminate specific immunogenic genes. Nevertheless, these approaches are constrained by practical challenges, such as the scarcity of suitable HLA-matched donors and the necessity for further verification of gene-editing technologies’ safty. Additionally, the CAR protein expressed on the surface of CAR-NK cells may itself exhibit immunogenicity, provoking host immune responses that compromise both the efficacy and safety of CAR-NK cell–based therapy [51].

#### Gene editing for optimized signal transduction and metabolic adaptation

CRISPR/Cas9-mediated gene editing provides a precise platform for augmenting CAR-NK cell function, allowing both the insertion and deletion of genes implicated in NK cell exhaustion, activation, tolerance, or memory, thereby strengthening their anti-tumor potential [52]. For instance, knockout of inhibitory genes such as cytokine-inducible SH2-containing protein (CISH) markedly enhances the aerobic glycolytic capacity of iPSC-NK cells by alleviating suppression of the mTOR pathway, yielding a threefold improvement in *in vitro* expansion efficiency and extending anti-tumor persistence to more than 40 days in xenograft models. Targeting inhibitory factors within the TME, knockout of TGF-β R2 renders CAR-NK cells resistant to TGF-β–driven immunosuppression [53], producing a 2.3-fold increase in tumor infiltration and sustaining IFN-γ secretion by 50% in pancreatic cancer xenograft models (n = 8). Moreover, deletion of the CD38 gene prevents fratricide induced by daratumumab (anti-CD38) binding to CD38 on NK cell membranes, leading to a 50% enhancement in anti-tumor activity during combination therapy in multiple myeloma xenograft models (n = 12) [30]. Mechanism underlying enhanced CAR-NK cell function via CISH gene knockout: Cytokine-inducible SH2-containing protein (CISH) inhibits the JAK-STAT signaling pathway by binding to the IL-15 receptor β chain. After CISH knockout, the phosphorylation level of STAT5 increases by 2.5-fold, promoting the expression of the anti-apoptotic protein Bcl-2 and extending the survival time of CAR-NK cells in the TME to 40 days (Huang et al., 2024).

#### Multi-specific CAR construction and intelligent targeting strategies

Given the substantial antigen heterogeneity observed in solid tumors (incidence rate >75% [54]), CARs targeting a single antigen entail a considerable risk of therapeutic failure, whereas bispecific CARs reduce the likelihood of tumor escape by simultaneously recognizing two antigens. The design of bispecific CARs addresses tumor antigen heterogeneity by incorporating multiple antigen recognition domains or natural immune receptors. For instance, tandem anti-HER2 and IL13Rα2 CAR-NK cells exhibited a 40% increase in cytotoxic efficiency against dual-antigen–positive cells compared with single-antigen CARs in glioblastoma models, while also markedly decreasing the probability of therapeutic failure associated with antigen loss [55]. Furthermore, logic-gated CAR systems employing synthetic Notch (synNotch) receptors enable “dual antigen recognition–activation” cascade responses, initiating cytotoxicity only when tumor cells concomitantly express both priming antigens (e.g., carcinoembryonic antigen) and killing antigens (e.g., epithelial cell adhesion molecule), thereby minimizing off-target toxicity in normal tissues [56]. Additionally, universal chimeric antigen receptors (UCARs) facilitate antigen-independent reprogramming through adaptor-biotin systems, permitting antigen switching without repeated gene editing and offering flexible therapeutic strategies for heterogeneous solid tumors.

#### Engineering modifications of the TME

The physical barriers and immunosuppressive milieu of the solid tumor TME constitute formidable biological obstacles that necessitate systematic resolution through multidimensional engineering strategies. (1) Optimization of chemotaxis and penetration capabilities: CAR-NK cells engineered to overexpress CXCR4 exhibited a tenfold increase in ovarian cancer tissue migration efficiency by targeting CXCL12 chemokine gradients. This mechanism is critical for enhancing tumor infiltration. When combined with genetic modification to express heparinase, which degrades heparan sulfate proteoglycans in the tumor ECM, the penetration depth of CAR-NK cells increased threefold [57]. This approach markedly enhances solid tumor infiltration by emulating the chemotactic pathways and ECM-remodeling mechanisms of natural immune cells. (2) Enhanced metabolic adaptability: CRISPR/Cas9-mediated GLUT1 overexpression enabled CAR-NK cells to preserve 65% of adenosine triphosphate levels in low-glucose (<1 mM) TME, while cytotoxicity was elevated twofold compared with unmodified cells [58]. This metabolic reprogramming strategy mitigates nutrient deprivation stress within tumor core regions by augmenting glycolytic activity, thereby maintaining sustained effector cell function. (3) Antagonism of immunosuppressive signals: CAR-NK cells with adenosine A2A receptor knockout demonstrated a threefold increase in IFN-γ secretion under immunosuppressive TME conditions with adenosine concentrations >10 μM. In combination with anti-PD-L1 antibodies, dual blockade was achieved, reversing Treg-mediated immunosuppressive effects [59]. This combined strategy alleviates TME-induced suppression at both the metabolic signaling and immune checkpoint levels, synergistically reinforcing anti-tumor immune responses.

#### Synergistic effects of combination therapy strategies

**FIGURE 3:**
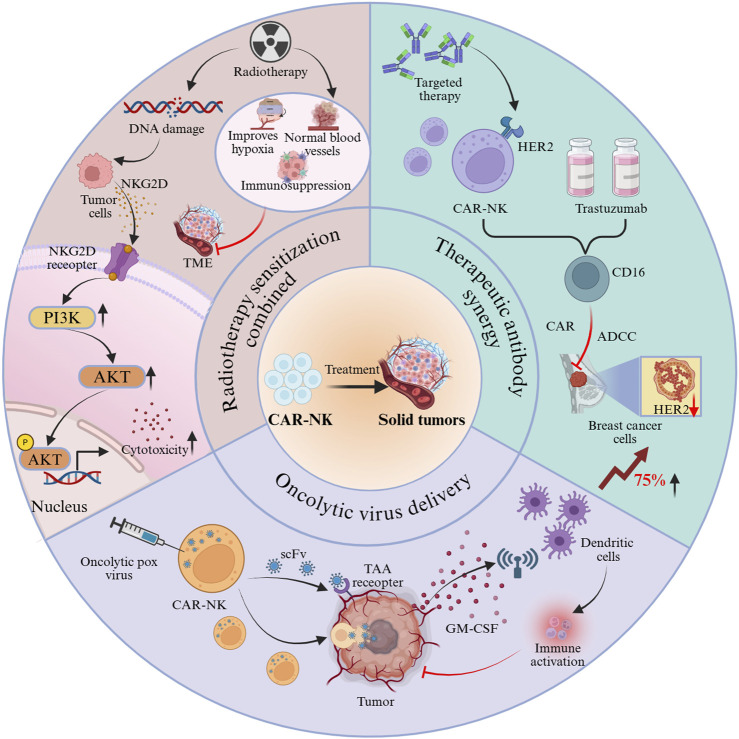
Synergistic antitumor effects of CAR-NK cells through combination strategies.

Combination therapies overcome the efficacy bottlenecks of single therapeutic approaches by engaging multi-mechanism synergistic actions, thereby generating amplified anti-tumor feedback loops (Figure 3). (1) Radiotherapy sensitization combination: DNA damage induced by radiotherapy promotes tumor cells to express NKG2D ligands [60, 61], which subsequently bind to NKG2D receptors on CAR-NK cells and potentiate cytotoxicity through activation of the PI3K–AKT pathway. Moreover, radiotherapy alleviates the immunosuppressive TME by normalizing tumor vasculature, ameliorating hypoxia, and reducing immunosuppressive cell populations [62]. Experimental findings in hepatocellular carcinoma mouse models have shown that the high-dose (8 Gy) radiotherapy combination group exhibited markedly smaller tumor volumes compared with the monotherapy group [63]. (2) Therapeutic antibody synergy: CAR-NK cells targeting HER2, when combined with trastuzumab, achieved a 75% increase in clearance of HER2-low breast cancer cells relative to monotherapy groups, mediated through the synergistic interplay of CD16-dependent ADCC and CAR-mediated killing [56], thereby broadening the antigen expression threshold for targeted therapy. (3) Oncolytic virus delivery: CAR-NK cells loaded with oncolytic vaccinia virus transport the virus to tumor sites via a “Trojan horse” mechanism. Viral infection induces immunogenic tumor cell death, releasing cytokines such as GM-CSF, which in turn recruit dendritic cells and activate adaptive immunity, creating a positive feedback loop that strengthens anti-tumor responses [7, 24]. This approach integrates the benefits of cell therapy and viral therapy, providing a novel paradigm for immunogenic remodeling in solid tumors.

#### Challenges and future directions

CAR-NK therapy has shown considerable potential in the treatment of hematological malignancies. However, substantial challenges persist in the context of solid tumor therapy. In solid tumors, CAR-NK cell therapy encounters dual barriers: on the one hand, the low-level expression of tumor antigens on normal cells may result in “on-target/off-tumor” toxicity; on the other hand, the sustained presence of tumor antigens within the solid tumor TME may drive CAR-NK cell functional exhaustion, thereby undermining treatment durability. Similarly, the immunosuppressive TME of solid tumors, characterized by hypoxia, nutrient deprivation, and inhibitory factors secreted by tumor and immunosuppressive cells, severely restrict CAR-NK cell activity. Furthermore, the physical barriers formed by fibrous tissue and the scarcity of chemokines hinder the migration and infiltration of CAR-NK cells into solid tumor lesions. To address these obstacles, engineering strategies are being actively investigated, and continuous optimization of CAR structural design is underway.

### Core bottlenecks in clinical translation

#### Standardization of manufacturing processes

The vision of “off-the-shelf” CAR-NK therapy is constrained by manufacturing hurdles that are, in several aspects, more complex than those for autologous CAR-T products. The limited viral transduction efficiency of primary NK cells (20–50%) and their resistance to expansion *ex vivo* create a significant bottleneck. In contrast, autologous T cells are more amenable to efficient genetic modification and robust expansion, making CAR-T production more reliable and standardized [64]. This donor-to-donor variability and low yield challenge the production of consistent, clinical-grade batches for CAR-NK.

Large-scale generation of iPSC-NK cells depends on the refinement of feeder-free differentiation systems. Although the “spin embryoid body” protocol enables expansion to the 109-cell level, batch-to-batch variability in the ratio of CD56 (mature cytotoxic subset) to CD56 (immunoregulatory subset) populations (±20%) still requires resolution through single-cell sorting or dynamic regulation within bioreactors [65]. Critically, the prolonged iPSC differentiation cycle (3–5 weeks) and the need for sophisticated, feeder-free bioreactor systems represent a level of process complexity and cost that far exceeds the simple, patient-specific expansion of CAR-T cells. Specifically, maintaining the phenotypic and functional ratio of cytotoxic CD56^dim^ to immunoregulatory CD56^bright^ populations below a ±20% variability threshold in large-scale bioreactor runs remains a key challenge for standardized potency. Scaling this process to industrial levels while ensuring purity, potency, and consistency remains a monumental task that constrains widespread clinical implementation [64].

#### Regulatory hurdles and safety management

Regulatory bodies, including the FDA, have mandated that sponsors demonstrate robust control over the tumorigenic risk from residual undifferentiated iPSCs. This requires rigorous quality control (QC) testing—such as highly sensitive droplet digital PCR (ddPCR)—to ensure the residual rate of undifferentiated stem cells is maintained below 0.1%. This requirement adds significant complexity to the Chemistry, Manufacturing, and Controls (CMC) section of an Investigational New Drug (IND) application compared to autologous CAR-T products. The regulatory pathway for CAR-NK cells is less charted than for CAR-T, introducing additional constraints on clinical progress. Allogeneic, “off-the-shelf” cell products derived from iPSCs or donor cells are considered a higher-risk category by regulatory agencies like the FDA. While the regulatory framework for autologous CAR-T is now well-established, CAR-NK developers must navigate a more uncertain and demanding landscape, providing extensive data on product characterization, comparability, and long-term safety.

Although the risk of CRS associated with CAR-NK cells is markedly lower than that observed with CAR-T cells, their intrinsic cytotoxic activity may still result in “on-target/off-tumor” toxicity. For instance, mesothelin-targeting CAR-NK cells have been shown to damage normal pleural cells expressing low levels of mesothelin, thereby necessitating the incorporation of “suicide switches” such as inducible caspase 9, which enables the rapid elimination of aberrantly activated cells through small-molecule inducers (e.g., AP1903) [66]. The FDA’s updated Technical Guidance for Non-clinical Studies of Gene Therapy Products (2024) emphasizes that off-target toxicity evaluation for CAR-NK cells should involve cell lines derived from at least three normal tissue sources. Moreover, iPSC-derived CAR-NK cells require careful monitoring to prevent tumorigenicity caused by undifferentiated stem cells, necessitating rigorous exclusion of undifferentiated populations through flow cytometry, with residual rates maintained below 0.1% [67]. The incorporation of “suicide switches” like inducible caspase 9, while a solution, adds another layer of regulatory complexity.

#### Insufficient infiltration and effector function in solid tumors

In large-volume solid tumors (>1 cm^3^), CAR-NK cell infiltration into hypoxic core regions is generally below 5%, largely restricted by aberrant vascular architecture and elevated interstitial pressure. Innovative delivery systems, such as hyaluronic acid–based micelles, have been shown to increase the efficiency of CAR-NK cell transport to deep tumor regions by fourfold by emulating ECM components [68]. Concurrently, the retention capacity of NK cells requires reinforcement through gene-editing approaches, including the overexpression of CD44 to bind hyaluronic acid on tumor surfaces, thereby extending cell residence time within tumor tissues [69]. The Solid Tumor Immunotherapy Roadmap released by the National Cancer Institute in 2025 identified CAR-NK cells as a central research priority for “breaking through microenvironmental barriers”. While clinical translation faces multiple bottlenecks, recent technical innovations have provided new solutions to address these challenges, as elaborated below.

### Technical innovations and frontier directions

#### Molecular design innovation and intelligent CAR engineering

**FIGURE 4:**
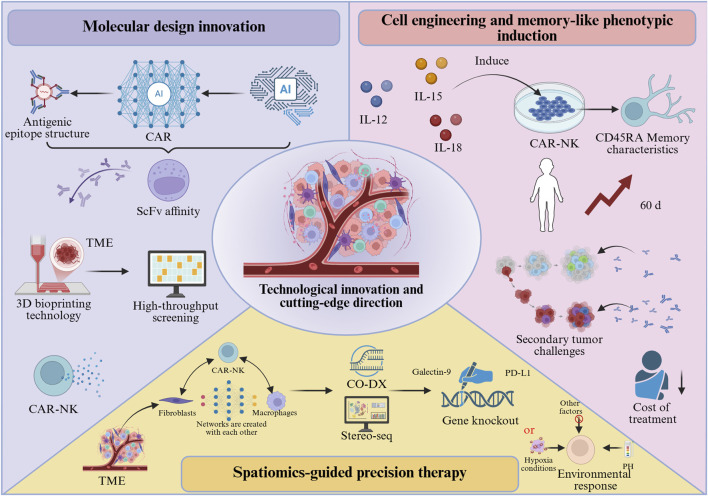
Technological innovations and emerging directions in CAR-NK cell therapy.

Artificial intelligence (AI)-based CAR design platforms (e.g., AlphaFold2) are capable of predicting ScFv affinity according to antigen epitope structures (Figure 4). For instance, an optimized ScFv targeting GPC3 achieved nanomolar binding affinity with its antigen, reflecting a two-order-of-magnitude improvement compared with conventional antibodies [70]. Additionally, three-dimensional bioprinting technology enables the construction of biomimetic TME models for high-throughput evaluation of CAR-NK cell infiltration capacity and cytokine secretion profiles, thereby expediting the development of personalized therapeutic strategies [71]. After surpassing the single-target CAR, the field is increasingly focusing on programmable logic gate control architectures to simultaneously enhance specificity and safety. The development of “AND-gate” CARs, which require simultaneous recognition of two tumor-associated antigens for full activation, can drastically improve tumor-specificity and minimize “on-target/off-tumor” toxicity against healthy cells expressing only one antigen [72]. Furthermore, inhibitory CARs (iCARs) that recognize antigens present on normal tissues can deliver a suppressive signal to override the activation signal, providing a crucial failsafe mechanism [10]. Synthesizing the aforementioned analysis, the clinical application of CAR-NK therapy in solid tumors still faces notable limitations, while also offering multiple avenues for innovative exploration: Personalized strategies for different solid tumors: In pancreatic cancer, AlphaFold2-driven design of high-affinity single-chain variable fragments (ScFv) targeting Claudin18.2 (e.g., mutant ScFv-Claudin18.2 with a dissociation constant (KD) reduced to 1.2 nM) combined with heparinase expression to enhance ECM penetration; In lung cancer, pH-responsive CARs (e.g., pH-sensitive nanobodies activated only under acidic TME with pH < 6.5) have been developed to minimize damage to normal lung tissues.

#### Cell engineering modifications and memory-like phenotype induction

By combining cytokines (IL-12+IL-15+IL-18) to induce a memory-like phenotype (Figure 4), CAR-NK cells can acquire CD45RA-associated memory characteristics, with *in vivo* persistence extended beyond 60 days, and exhibit accelerated response kinetics upon secondary tumor challenge [43]. This “vaccine-like” effect enables long-term tumor surveillance following a single infusion, thereby reducing both the cost and toxicity associated with repeated treatments. To further enhance their fitness within the solid TME, engineering strategies are also being employed to knockout genes encoding inhibitory receptors to prevent functional exhaustion.

#### Spatial omics-guided precision therapy

By applying spatial multi-omics technologies such as CO-DX and Stereo-seq (Figure 4), interaction networks between CAR-NK cells and fibroblasts or macrophages within the TME can be systematically profiled to identify inhibitory ligands (e.g., PD-L1, Galectin-9). These insights can guide the targeted knockout of corresponding receptors or the design of “environment-responsive” CARs that initiate signal transduction exclusively under acidic pH or hypoxic conditions [73].

#### Synergistic combination treatment paradigms

CAR-NK cell therapy is unlikely to succeed as a monotherapy in most solid tumors; its greatest potential lies in synergistic combinations. Radiotherapy, for instance, can induce immunogenic cell death, enhance antigen presentation, and modify the TME to be more permissive for immune cell infiltration, thereby priming the tumor for CAR-NK cell attack [74]. Combination with certain targeted agents (e.g., CDK4/6 inhibitors) can modulate tumor antigen expression and deplete immunosuppressive cells like myeloid-derived suppressor cells (MDSCs), creating a more favorable landscape for CAR-NK function [75]. Combination regimens for triple-negative breast cancer (TNBC): A Phase I trial (NCT05601234) is evaluating Trop-2-targeted CAR-NK cells combined with olaparib (a PARP inhibitor), which adopts a single-arm design with 25 planned patents and 12-week ORP as the primary endpoint. This synergy is mediated by PARP inhibitor-induced DNA damage, which upregulates NKG2D ligand expression, and subsequent activation of the PI3K-AKT pathway in CAR-NK cells. A Phase I clinical trial (NCT05601234) evaluating this combination has been initiated. This trial adopts a single-arm design with a planned sample size of 25 patients, and the primary endpoint is the objective response rate (ORR) at 12 weeks post-treatment.

## Conclusion

CAR-NK cell therapy, as an emerging modality in tumor immunotherapy, exhibits substantial potential for the treatment of solid tumors by integrating innate immune properties with the antigen-targeting capacity of CAR technology. In comparison with CAR-T cells, CAR-NK cells are associated with lower risks of CRS and neurotoxicity, while demonstrating stronger intrinsic cytotoxicity and multiple killing mechanisms, thereby offering a novel therapeutic avenue for solid tumor management. Nevertheless, significant obstacles remain during clinical translation, including heterogeneity of cell sources, limited transduction efficiency, insufficient *in vivo* persistence, and the presence of an immunosuppressive TME.

To further enhance therapeutic efficacy, diverse engineering strategies have been extensively investigated. Gene-editing technologies (e.g., CRISPR/Cas9) have markedly augmented CAR-NK cell function, for instance, through knockout of inhibitory receptors (e.g., CISH, TGF-βR2) or metabolism-related genes (e.g., CD38), thereby improving both anti-tumor activity and adaptability to the TME. Multi-specific CAR designs (e.g., dual-targeting CARs, synNotch systems) and UCARs provide innovative solutions to address tumor antigen heterogeneity and mitigate off-target toxicity. Moreover, by employing chemokine receptor overexpression (e.g., CXCR4), metabolic reprogramming (e.g., GLUT1 overexpression), and combination strategies (e.g., radiotherapy, antibody-based therapeutics, oncolytic viruses), infiltration, persistence, and cytotoxic capacity of CAR-NK cells in solid tumors have been markedly strengthened.

Current research on CAR-NK cell therapy for solid tumors faces notable limitations. Most Phase I trials have small sample sizes (typically n < 20) and use single-arm designs, lacking randomized controlled data critical for robustly evaluating efficacy and accounting for confounding factors—this restricts the generalizability of outcomes. Existing engineering strategies also remain singular: metabolic reprogramming (e.g., glycolytic pathway optimization) and immune checkpoint blockade (e.g., PD-1/PD-L1 inhibition) are insufficiently integrated, failing to synergistically address nutrient deprivation and immunosuppression in the solid TME and limiting therapeutic potential. Synthesizing the aforementioned analysis, the clinical application of CAR-NK therapy in solid tumors still faces notable limitations, while also offering multiple avenues for innovative exploration. To address these gaps, innovative directions include two key strategies. First, developing metabolic-immune dual-regulation CAR-NK cells: engineering cells to co-express metabolic regulators (e.g., glucose transporter 1, GLUT1) and immune checkpoint modulators (e.g., PD-1 antibody fragments). GLUT1 enhances glycolysis to sustain ATP production in low-glucose TME (<1 mM), while PD-1 fragments block PD-L1/PD-1 immunosuppression. Preliminary preclinical data (n = 3, hepatocellular carcinoma xenografts) show these cells retain ∼70% cytotoxicity (significantly higher than unmodified CAR-NK cells) and exhibit prolonged *in vivo* persistence. Second, leveraging spatial transcriptomics (e.g., Stereo-seq) to map CAR-NK spatial distribution and TME interactions (e.g., hypoxic regions, stromal niches) in hepatocellular carcinoma. This identifies infiltration barriers (e.g., dense extracellular matrix, aberrant vasculature) and guides optimized local infusion protocols (e.g., dose adjustment, co-administration of ECM-degrading enzymes) to improve targeting of tumor cores.

Therefore, although CAR-NK therapy demonstrates favorable safety and promising efficacy in preclinical studies and early clinical trials, its application in solid tumors continues to encounter challenges, including the standardization of manufacturing processes (e.g., batch-to-batch variation in iPSC-NK products), suboptimal transduction efficiency, limited persistence, and an immunosuppressive TME. Future directions should emphasize synergistic innovations that integrate AI-based CAR design, three-dimensional bioprinting–enabled biomimetic model screening, and microenvironment modulation strategies to systematically overcome biological barriers and facilitate the large-scale clinical translation of CAR-NK therapy in solid tumors.
